# The DNA Vaccines for the Gn and Gc Heterologous Polymer of Severe Fever with Thrombocytopenia Syndrome Virus Induce Potent Immunogenicity in Mice

**DOI:** 10.3390/vaccines13121186

**Published:** 2025-11-24

**Authors:** Qiuju He, Xiaojuan Liu, Jincheng Tong, Huan Li, Heng Zhang, Jiamin Chen, Mengyi Zhang, Zhihua Li, Qianqian Li

**Affiliations:** Institute of Medical Biology, Chinese Academy of Medical Sciences & Peking Union Medical College, Kunming 650118, China; s2023018003@pumc.edu.cn (Q.H.); liuxj0423@163.com (X.L.); tongjc327@outlook.com (J.T.); lihuan@bjmu.edu.cn (H.L.); 15198704875@163.com (H.Z.); charming20251030@163.com (J.C.); zhangmengyi1001@163.com (M.Z.)

**Keywords:** SFTSV, Gn, Gc, DNA vaccine, humoral response, cellular response

## Abstract

Introduction/Background: Severe fever with thrombocytopenia syndrome virus (SFTSV) poses a threat to global public health with a mortality rate of up to 30%. However, there is currently no commercialized SFTSV vaccine. This study focused on the construction of DNA vaccines with different structures based on the surface glycoproteins Gn and Gc to identify the immunodominant conformations. Methods: The DNA vaccines encoding secretory proteins including Gn or Gc monomer, heterodimer of Gn and Gc (dimer), two forms of hexamer composed of the Gn and Gc heterodimer (hexamer-1 and hexamer-2) or ferritin nanoparticles of Gn, and non-secretory proteins including Gn (Gn-TM) and Gc (Gc-TM) were constructed. Western blot confirmed the expression level and the specificity of those DNA vaccines. After vaccinating mice with those DNA vaccines, its induced humoral and cellular immunity were comprehensively evaluated. Results: The DNA vaccines were constructed successfully. The DNA vaccines of Gn and polymers including dimer, hexamer-2, and ferritin nanoparticles inducing stronger binding antibody, neutralizing antibody, and antibody-dependent cellular cytotoxicity (ADCC) activity. The neutralizing antibody induced by these constructs was also cross-recognized by other five SFTSV pseudovirus strains. However, the T cell response induced by Gc, dimer or hexamer-2 DNA vaccines were significantly higher than those in most other groups, including Gn. Conclusion: The DNA vaccines encoding dimer or hexamer-2 demonstrated superior immunogenicity over other conformations, after taking the results of humoral and cellular responses into account. This study revealed the advantages of using polymer conformations in SFTSV vaccine design and provided new targets in SFTSV vaccine development.

## 1. Introduction

Severe fever with thrombocytopenia syndrome (SFTS) is a naturally occurring infectious disease caused by the severe fever with thrombocytopenia syndrome bunyavirus (SFTSV), which is transmitted via tick vectors and was first reported in China in 2009 [1,2,3]. The disease is endemic to East and Southeast Asia (China, Japan, South Korea, Thailand) with expanding transmission foci, including detection in central Beijing’s urban parks in 2024 [4]. China reports the highest incidence (<0.1–4.2 cases/100,000 population nationally, peaking at 127.6 cases/100,000 in hotspots), most infections occurring between April and December in elderly farmers and veterinarians, and case fatality rates ranging from ~28.6% (severe cohorts in Anhui, China) to 12–50% globally (linked to advanced age and delayed care) [5,6]. The primary vector, Haemaphysalis longicornis (including parthenogenetic lineages), has 1.86% SFTSV RNA prevalence in endemic-area ticks (vs. 0.41% elsewhere); human seroprevalence reaches 0.3–20.2% in China (3.0–4.8% on coastal islands) and 0.2–5.9% in South Korea, while close contacts of cases show 5.59% RNA positivity (indicating human-to-human transmission risk). Carnivores (6.34% seroprevalence) and hedgehogs (high seropositivity) are served as the key viral reservoirs [7,8]. The clinical manifestations of SFTS are fever, gastrointestinal discomfort, reduced platelet count and in severe cases, pancreatic injury, myocardial damage, central nervous system abnormalities, and death [6]. The general population is broadly susceptible. Due to the absence of specific vaccines or antiviral drugs for SFTS, the mortality rate among patients with SFTS remains around 30%, with even higher fatality rates observed among immunocompromised individuals and the elderly [5,6,9,10]. Due to its expanding geographic range and the lack of commercial vaccines or specific antivirals, SFTSV’s six identified genotypes (A–F) exhibit pathogenicity, with cross-genotype infection risks further complicating control, underscoring the urgency of effective vaccine development [11].

SFTSV belongs to the order Bunyavirales, family Phenuiviridae, and genus Phlebovirus [12]. It is a negative-strand RNA virus with a genome of approximately 11,500 nucleotides in total length. The genome consists of three segments: L, M, and S [13]. Its main antigen proteins of Gn and Gc located on the viral surface are encoded by M segment, which primarily translates as a precursor Gp protein and is then cleaved by proteases to produce Gn and Gc. Gn and Gc mediate the infection of host cells through attachment and fusion [14]. The two proteins form a heterodimer, and multiple dimers are tightly arranged on the viral surface, embedding into each other through lateral interactions, further forming viral envelope pentamers and hexamers, stabilizing the pre-fusion structure of the viral particle [15]. An ordered network formed by those pentamers and hexamers creates an icosahedral glycoprotein shell [16]. After virus recognition of host cells, the heterodimeric depolymerizes and releases Gc. As a type II membrane fusion protein, Gc then forms a hairpin trimer to promote membrane fusion [17]. Gn and Gc are the main targets for specific neutralizing antibodies. Notably, the aromatic residue F699 on the fusion loop of the Gn and Gc heterodimer serves as a specific neutralizing antibody recognition site [18].

Existing studies have confirmed that Gn and Gc are key targets for inducing protective immunity. However, the Gn and Gc proteins form the complex structure conformations on the virus surface, and the effect of these different structures on inducing immune responses remain unclear, such as their oligomeric conformations (e.g., heterodimers, hexamers). In addition, the regulatory mechanism of different polymeric antigen forms on the immune response remains unclear, and there is a lack of systematic immune evaluation for oligomeric antigens. Our previous study preliminarily found that the effect of Gn or Gc trimer on inducing neutralizing antibody and eliminating pseudovirus in vivo is superior to their corresponding monomers [19], demonstrating the importance of applying structured conformational design in the studies of SFTSV vaccine. In order to quickly screen for more available protein conformations, a DNA vaccine platform was utilized in the present study for its advantages such as stability and short development cycles [20].

In this study, DNA vaccines encoding different conformations (monomer, heterodimer, two types of heterohexamers, ferritin nanoparticles) based on the natural structural characteristics of SFTSV Gn/Gc were designed and constructed. Specifically, the CAT and T4 motifs were used to construct the heterohexamer conformations [21,22]. By verifying the antigen expression efficiency in vitro, combined with in vivo evaluation of humoral immunity (binding antibodies, neutralizing antibodies, ADCC activity) and cellular immunity (Th1/Th2-type immune responses), this study screened antigen conformations with superior immunogenicity, thereby providing an experimental basis for the optimization of antigen design in SFTSV vaccines.

## 2. Materials and Methods

### 2.1. Cells, Pseudoviruses, and Proteins

HEK-293F cells, HEK-293T cells, Huh7 cells, SFTSV pseudoviruses of the six genotypes, and SFTSV antigen proteins Gn and Gc used for ELISA coating and ELISPOT stimulation are all preserved by our laboratory.

### 2.2. Main Reagents and Instruments

PBS was obtained from ServiceBio (Wuhan Servicebio Technology Co., Ltd., Wuhan, China); FBS was obtained from TransGen (TransGen Biotech, Beijing, China); DMEM was obtained from Gibco (Thermo Fisher Scientific (Suzhou) Instruments Co., Ltd., Suzhou, China); Mouse lymphocyte separation medium was obtained from Dakewei (Dakewe Biotech Co., Ltd., Shenzhen, China); HRP-conjugated goat anti-mouse IgG was obtained from Thermo (Thermo Fisher Scientific Inc., Waltham, MA, USA); Mouse IFN-γ ELISPOTPLUS (ALP) kits and Mouse IL-4 ELISPOTPLUS (ALP) kits were obtained from Mabtech (Mabtech AB, Nacka Strand, Sweden); A plate washer was obtained from Tuopu (Beijing Tuopu Analytical Instruments Co., Ltd., Beijing, China); A high-throughput multimode microplate detection system was obtained from PerkinElmer (REVVITY, Waltham, MA, USA); fluorescence ELISPOT reader was obtained from CTL (Cellular Technology Ltd., Shaker Heights, OH, USA); 96-well EIA/RIA plates were obtained from Corning (Corning Incorporated, Corning, NY, USA).

### 2.3. DNA Vaccines Design and Construction

**Figure 1:**
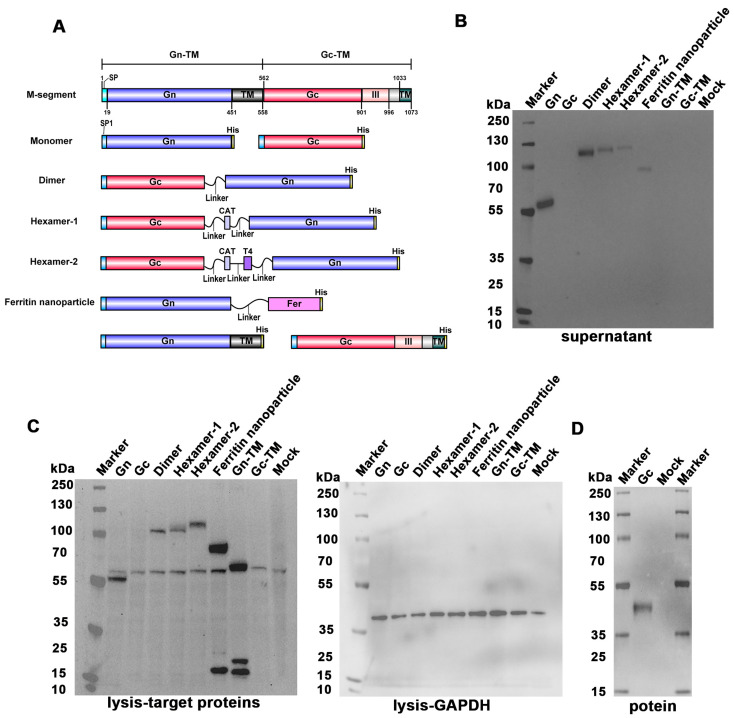
The design and Western blot validation of the SFTSV DNA vaccines. (**A**) The protein region of the M segment of HB29 strain. The fragment of Gn or Gc from M-segment were cloned into vector pCDNA3.1(+) to construct DNA vaccines. The M segment corresponds to the full-length M segment of HB29. Both SP and SP1 are signal peptides, and the region from 996 to 1033 corresponds to the stalk region sequence of SFTSV. Monomers: truncated Gn and Gc, respectively; Dimer: linked secretory Gn and Gc; Hexamer-1: constructed with CAT for linkage; Hexamer-2: constructed with CAT+T4 for linkage; Ferritin nanoparticles: connected secretory Gn and ferritin. Constructs in the last row: full-length Gn and Gc, respectively. (**B**–**D**) Western blot analysis of the supernatant (**B**), cell lysate (**C**), and purified protein (**D**) after transfection. (**C**) Anti-His antibody was used to detect the Gn or Gc proteins, and anti-GAPDH antibody was used to detect the GAPDH which served as a loading control protein. The supernatant and the cell lysate were obtained 72 h after transfecting the plasmids of DNA vaccines into HEK-293F cells.

The Gn and Gc DNA sequences of HB29 strain were obtained from GenBank (access number: HM745931), and synthesized according to the design in Figure 1A. The segment of aa19-451 and aa562-901 was used in secretory type of Gn and Gc, respectively, and full-length sequences of aa19-558 and aa562-1073 were used in non-secretory type of Gn and Gc, respectively. Subsequently, we constructed multimers using the secretory forms of Gn and Gc. Dimer of Gn and Gc was linked by linker directly. Heterologous hexamers of Gn and Gc such as hexamer-1 and hexamer-2 were constructed using trimeric motifs of CAT or CAT combined with T4. At the same time, the ferritin was linked to Gn to form ferritin nanoparticles. In addition, a His-tag was appended to each construct for subsequent characterization, with GGGGSGGGGS as the linker. These sequences were cloned into the pcDNA3.1(+) vector and sequencing confirmation was performed after monoclonal bacterial colony amplification and plasmids extraction.

### 2.4. Western Blot

The recombinant plasmids were transfected into HEK-293F cells using the JETPRIME transfection reagent (Polyplus, Illkirch-Graffenstaden, France). After 72 h, the supernatant and cell lysates were collected for Western blot analysis. 10 μg of total protein from cell lysates or 10 μL of supernatant were mixed with loading buffer (YamayBio, Shanghai, China) and incubated at 95 °C for 10 min, followed by electrophoresis on a 12% SDS-PAGE gel for 45 min. The proteins were then transferred onto a PVDF membrane and blocked with 5% skim milk for 2 h at room temperature. HRP-conjugated anti-his antibody (ACROBiosystems, Beijing, China) (1:5000) was diluted in TBST buffer (10 mM Tris-HCl, pH 8.0, 150 mM NaCl, and 0.1% Tween 20) and incubated with the membrane at 37 °C for 2 h. After washing the membrane with TBST four times (10 min per wash), an ECL luminescent solution (Milippore, Burlington, MA, USA) was added and the signal was visualized using a fully automated chemiluminescence imaging system.

### 2.5. The Feed and Vaccination of Experimental Animals

Specific pathogen-free (SPF)-grade female BALB/c mice, aged 6–8 weeks and weighing 18–22 g, were purchased from Beijing SiPeifu Biotechnology (Beijing, China), with the animal qualification certificate number: SCXK 2024-0001. All experimental procedures were approved by the Animal Welfare and Ethics Committee of the Institute of Medical Biology, Chinese Academy of Medical Sciences, under the review number: DWSP202408032. The mice were randomly divided into nine groups (10 mice per group) and vaccinated intramuscularly with the DNA vaccines followed by electrical stimulation at 0, 2, 4, and 24 weeks. The first three vaccinations administered 50 μg per dose, while the fourth vaccination used 100 μg. Blood samples were collected as described in corresponding sections.

### 2.6. Neutralizing Antibody Detection by Pseudovirus System

The pseudovirus detection experiments were performed in a P2 laboratory. Threefold serially diluted mouse sera, starting at a 1:30 dilution, were added to white 96-well plates. Then, 50 µL of SFTSV pseudovirus (1 × 10^4^ TCID50/mL) were added in each well, then mixed gently and incubated at 37 °C in a 5% CO_2_ incubator for 1 h. Next, 100 µL of cell suspension containing Huh-7.0 cells (3 × 10^5^ cells/mL) was added to the 96-well plates and incubated at 37 °C in a 5% CO_2_ incubator for 48 h. Subsequently, 50 µL of luciferase detection reagent (PerkinElmer, Waltham, MA, USA) was added in each well, and the luminescence signal was read by a high-throughput multimode microplate detection system.

### 2.7. Enzyme-Linked Immunosorbent Assay (ELISA)

The protein Gn or Gc was coated in 96-well plates at a final concentration of 2 µg/mL and incubated at 4 °C overnight. After discarding the coating solution and washing twice by PBST (PBS buffer containing 0.05% Tween 20), the plates were blocked with PBST containing 1% BSA at 37 °C for 1 h. The serum samples were initially diluted at 1:200 and then used to perform a 2-fold serial dilution, and 100 µL of each dilution was added to each well. After incubating at room temperature for 1 h, the plates were washed by PBST for four times. The secondary antibody of HRP-conjugated goat anti-mouse IgG (diluted 1:10,000 in PBS contain 0.1% BSA) was added at 100 µL/well and incubated at room temperature for 1 h. After washing the plates for four times, 100 µL per well of TMB substrate was added and incubated in the dark at room temperature for 5–10 min for color development. Finally, the reaction was stopped by adding 50 µL per well of stop solution (2 M H_2_SO_4_) and the absorbance at 450 and 630 nm was measured by a microplate reader. The cut-off value was defined as 2.1 times the mean value of negative control group.

### 2.8. IFN-γ and IL-4 Enzyme-Linked Immunosorbent Assay (ELISpot)

The isolation of the mice splenic lymphocytes was conducted by using a mouse lymphocyte separation medium (Dakewei, Shenzhen, China) following the manufacturer’s instructions. Mouse IFN-γ ELISPOTPLUS (ALP) kits and Mouse IL-4 ELISPOTPLUS (ALP) kits were used to detect the IFN-γ and IL-4 secreted T cells. The pre-coated ELISpot 96-well plates were washed four times with sterile PBS at 200 μL per well. Then, 200 μL per well of ELISpot-specific serum-free medium was added and incubated at room temperature for at least 30 min. The stimuli of Gn or Gc proteins were diluted to 20 μg/mL and added at 50 μL per well with 50 μL of culture medium as a negative control in wells without stimulus; then, 50 μL of cell suspension was added to each well. The contents of splenic lymphocytes were 3 × 10^5^ cells per well for IFN-γ and 7 × 10^5^ cells per well for IL-4 detection plates. The plates were cultured in a 37 °C 5% CO_2_ incubator for 40 h. After discarding the cell suspension, 200 μL of pre-chilled sterile deionized water was added per well for 2 min of incubation. Following removal of the deionized water, the plates were washed five times with 200 μL PBS per well with 2 min incubation for each wash. IFN-γ (1:1000) or IL-4 (1:1000) primary antibodies were diluted in PBS containing 0.5% bovine serum and added to the corresponding ELISpot plates at 100 μL per well. After incubation at room temperature for 2 h, the plates were washed as described above. Secondary antibodies of ALP-labeled streptavidin (1:1000) were diluted in PBS containing 0.5% bovine serum and added to the corresponding ELISpot plates at 100 μL per well. After incubation at room temperature for 1 h, the plates were washed five times. The chromogenic BCIP/NPT-plus solution was filtered through a 0.45 μm filter, and 100 μL was added to each well. Plates were incubated in the dark at 37 °C for 2–5 min, or until distinct spots appear in positive wells. Then, the chromogen solution was discarded and the plates were washed by deionized water. After drying the plates, an ELISpot plate reader was used to measure the plots. Final data is expressed as spot-forming units (SFUs).

### 2.9. Antibody-Dependent Cellular Cytotoxicity (ADCC) Response

The method of ADCC assay for detecting sera from SFTSV-vaccinated mice was established in our previous study [19]. The Jurkat-Fc*γ*RIII-NFAT-Luc reporter cells were used as the effector cells. Mouse sera performed a 4-fold gradient dilution starting at a 1:30 dilution. The preparation and verification of target cells containing Gn and Gc proteins, the addition of the antibody, target and effector cells, and the detection of the Fluorescence values were all followed by our previous study [19].

### 2.10. Statistical Analysis

Statistical analysis was performed using GraphPad Prism (v8.0.1, Build 224). One-way ANOVA was employed to analyze inter-group differences, with *p* < 0.05 considered statistically significant.

## 3. Results

### 3.1. Design and Construct the SFTSV DNA Vaccines

The design of the SFTSV DNA vaccines based on Gn and Gc are shown in Figure 1A. According to our previous study, the complete sequences of secretory Gc did not express and the segment without domain III were expressed successfully. Hence, the same segment of Gc were employed in this study. The transmembrane (TM) regions were removed in the DNA vaccines encoding secretory protein including Gn and Gc monomer (group Gn and Gc), heterodimer of Gn and Gc (group dimer), the ferritin nanoparticles of Gn (group ferritin nanoparticles), and the trimers of the Gn and Gc heterodimer, which were constructed by using trimeric motif of CAT (group hexamer-1) or CAT plus T4 (group hexamer-2). In order to compare the immunogenicity of secretory protein with non-secretory protein, the DNA vaccines encoding non-secretory Gn or Gc were constructed (group Gn-TM and Gc-TM).

### 3.2. Validation of the DNA Vaccines In Vitro

All plasmids were validated by DNA sequencing. The theoretical molecular weights of Gn, Gc, dimer, hexamer-1, hexamer-2, ferritin nanoparticles, Gn-TM, and Gc-TM were 50 kDa, 39 kDa, 87 kDa, 270 kDa, 279 kDa, 1704 kDa, 59 kDa, and 56 kDa, respectively. Western blot analysis of the target protein and Glyceraldehyde-3-phosphate dehydrogenase (GAPDH) (internal reference) in cell lysates was conducted using two separate membranes with the same sample. This setup prevents antibody cross-interference, and ensures the specificity of signals and accuracy of quantification. The specific protein bands of those DNA vaccines were all detectable in both supernatant and cell lysate, except for those derived from the plasmids of Gc and Gc-TM after transfection (Figure 1B,C). However, due to the protein depolymerization in denaturing electrophoresis system, the apparent molecular weight of all polymeric forms corresponded to that of their monomeric subunits. The Gc protein in supernatant was detectable in Western blot validation following purification (Figure 1D), indicating its low expression level in supernatant. The undetectable Gc-TM protein bands may be attributed to unsuccessful expression of the full-length Gc protein.

### 3.3. The Humoral Response of the SFTSV DNA Vaccines In Vivo

**Figure 2:**
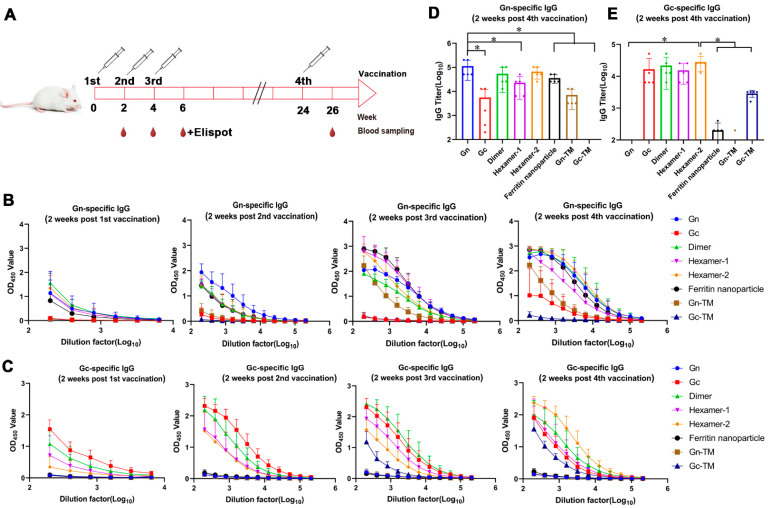
The binding antibody induced by SFTSV DNA vaccines. (**A**) BALB/c mice were injected intramuscularly with DNA vaccines followed by electrical stimulation at 0, 2, and 4 weeks (50 μg/mice) and at 24 weeks (100 μg/mice). Mice injected with PBS were served as a mock group. Blood samples were obtained two weeks after each vaccination to detect the binding and neutralizing antibody. (**B**,**C**) ELISA analysis of the Gn (**B**) or Gc (**C**)-specific IgG titers at two weeks after the first, second, third, and fourth vaccinations. (**D**,**E**) The statistical analysis of the Gn (**D**) or Gc (**E**)-specific IgG titers in the sera samples of mice at the 200 fold dilution at two weeks after the fourth vaccination. *, significantly different between the groups indicated in the figures (*p* < 0.05) as determined by one-way ANOVA. Data are presented as mean ± SD (error bars represent SD, *n* = 5 mice per group).

BALB/c mice were intramuscularly injected with 50 μg of DNA vaccines at 0, 2, and 4 weeks and 100 μg at 24 weeks follow by electrical stimulation, and blood samples were obtained 2 weeks after each vaccination to detect the binding and neutralizing antibody (Figure 2A).

The humoral immune responses induced by those DNA vaccines were evaluated by measuring the binding antibody, neutralizing antibody, and ADCC activity. The two main antigen Gn- or Gc-specific binding antibody were tested by ELISA (Figure 2B–E). In most groups, binding antibodies specifically recognizing Gn or Gc presented positive levels after the first vaccination and increased with the number of immunizations (Figure 2B,C). For the Gn-specific IgG, after the first immunization, the initial average OD values of the Gn, Dimer, Hexamer-1, Hexamer-2, and Ferritin nanoparticle groups were approximately 1. Following the second immunization, the initial average OD value of the Gn group increased to 1.93, while those of the other groups rose to around 1.5. Furthermore, after the third and fourth immunizations, the binding antibody levels of the Hexamer-1, Hexamer-2, and Ferritin nanoparticle groups were effectively enhanced, with their initial average OD values all approaching 3 (Figure 2B). Specifically regarding Gc antigen-specific binding antibodies (Figure 2C), a similar trend was observed: after the first immunization, only the Gc and Dimer groups had initial average OD values above 1.0. However, after the second, third, and fourth immunizations, the average OD values of the Gc, Dimer, Hexamer-1, and Hexamer-2 groups all increased to approximately 2.

After statistical analysis of the results of the fourth vaccination, group Gn, dimer, and hexamer-2 induced higher IgG titers (Gn-specific IgG for Gn and ferritin nanoparticles groups and Gn- or Gc-specific IgG for groups of Gn-Gc polymer) than other groups in the average level and there were no significant statistical difference between those three groups (Figure 2D,E). The geometric mean titers of binding antibodies (Gn-specific) in these three groups were 112,640, 53,760, and 66,560, respectively (Figure 2D). In addition, the Gn group exhibited significantly higher titers than the Hexamer-1, Ferritin nanoparticle, and Gn-TM groups. For Gc-specific binding antibodies, The Gc, Dimer, Hexamer-1, and Hexamer-2 groups induced higher IgG titers than other group in the average level, and the geometric mean titers of binding antibodies in these four groups were 16,640, 21,760, 15,360, and 25,600, respectively (Figure 2E).

**Figure 3:**
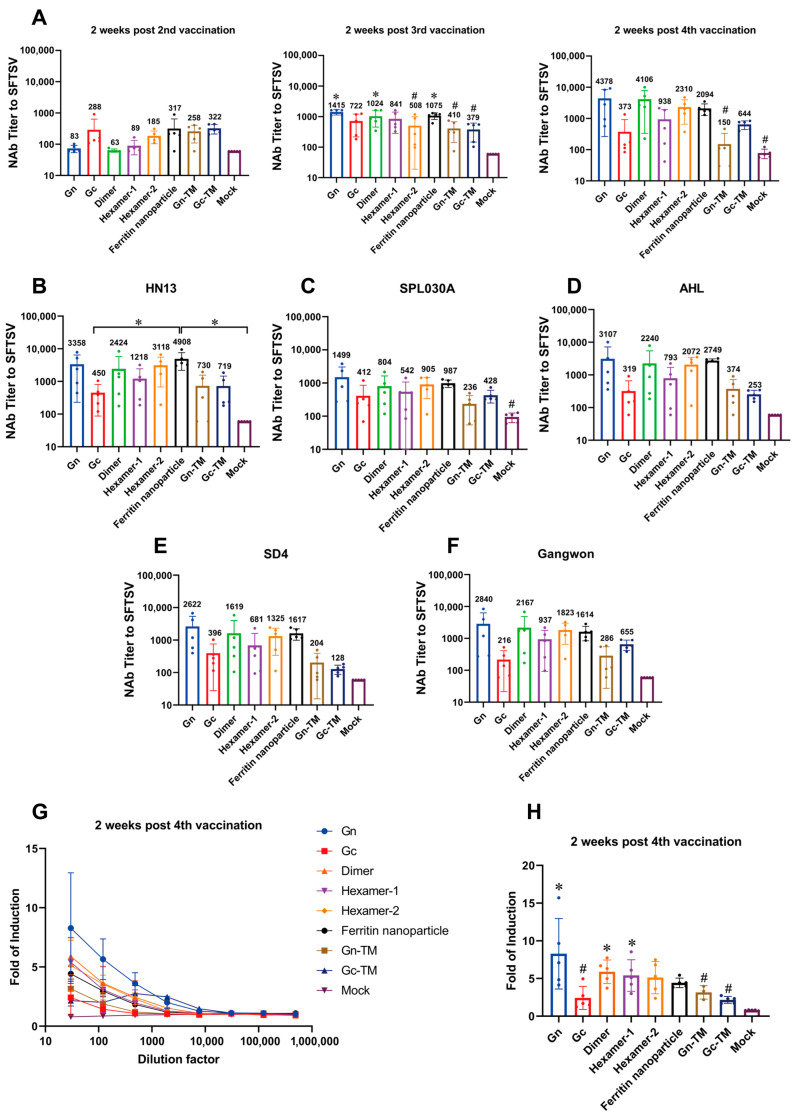
The neutralizing antibody titer and ADCC activity induced by the DNA vaccines. (**A**) The neutralizing antibody titer against SFTSV HB29 strain detected by pseudovirus system at two weeks after the second, third, and fourth vaccinations. (**B**–**F**) The neutralizing antibody titer against SFTSV HN13 (**B**), SPL030A (**C**), AHL (**D**), SD4 (**E**), and Gangwon (**F**) strains detected by the pseudovirus system at two weeks after the fourth vaccination. (**G**) The ADCC activity induced by the DNA vaccines at two weeks after the fourth vaccination at the 30–491, 520-fold dilution of the sera samples. (**H**) The statistical analysis of the ADCC activity induced by the DNA vaccines at the 30-fold dilution of the sera samples. Mice were treated as described in Figure 2A. *, significantly different from the mock group (**A**,**H**) or between the groups indicated in the figure (**B**) (*p* < 0.05); #, significantly different from Gn group (**A**,**H**) (*p* < 0.05) as determined by one-way ANOVA. Data are presented as mean ± SD (error bars represent SD, *n* = 5 mice per group).

The SFTSV pseudovirus containing Gn and Gc glycoproteins was used to detect the neutralizing antibody titer (Figure 3A–F).

The DNA vaccine elicited relatively high levels of neutralizing antibodies against the pseudovirus HB29 (type D) in mice following the second, third, and fourth immunizations (Figure 3A). After the third immunization, the Gn, Dimer, and Ferritin nanoparticle groups had significantly higher neutralizing antibody levels compared to the mock group, with respective levels of 1415, 1024, and 1075. Notably, the Gn group showed distinct neutralizing antibody titers from the Hexamer-2, Gn-TM, and Gc-TM groups. This indicates that the Gn, Dimer, and Ferritin nanoparticle constructs effectively induced high neutralizing antibody titers in mice after the third immunization. Furthermore, after the fourth immunization, the neutralizing antibody titers of the Gn, Dimer, Ferritin nanoparticle and hexamer 2 groups reached as high as 4378, 4106, 2094, and 2310, respectively—higher than other groups on the average level.

Furthermore, the neutralizing antibody induced by these four groups also cross-recognized and neutralized other five SFTSV pseudovirus strains at higher average level, including HN13 (type A), SPL030A (type B), AHL (type C), SD4 (type E), and Gangwon (type F). In HN13 strain test, the ferritin nanoparticles group’s titer was significantly higher than those of Gc and mock groups (Figure 3B–F).

In ADCC assay, after the fourth vaccination, the ADCC activity induced by groups of Gn, dimer, and hexamer-1 was significantly higher than that in the mock group, with the Gn group also showing significantly higher activity than Gc, Gn-TM, and Gc-TM groups (Figure 3G,H). Specifically, the ADCC induction fold of the Gn, Dimer, and Hexamer-1 groups reached as high as 8.28, 5.89, and 5.40, respectively.

Notably, the binding antibody, neutralizing antibody, and ADCC activity induced by Gn were all significantly higher than those induced by Gn-TM (Figure 2D and Figure 3A,H), demonstrating the effective enhancement of immunogenicity by using secretory protein-expressed plasmid in DNA vaccine design. In summary, the DNA vaccines of Gn, dimer, hexamer-2, and ferritin nanoparticles showed superiority in inducing humoral immunity.

### 3.4. The Cellular Response of the SFTSV DNA Vaccines In Vivo

**Figure 4:**
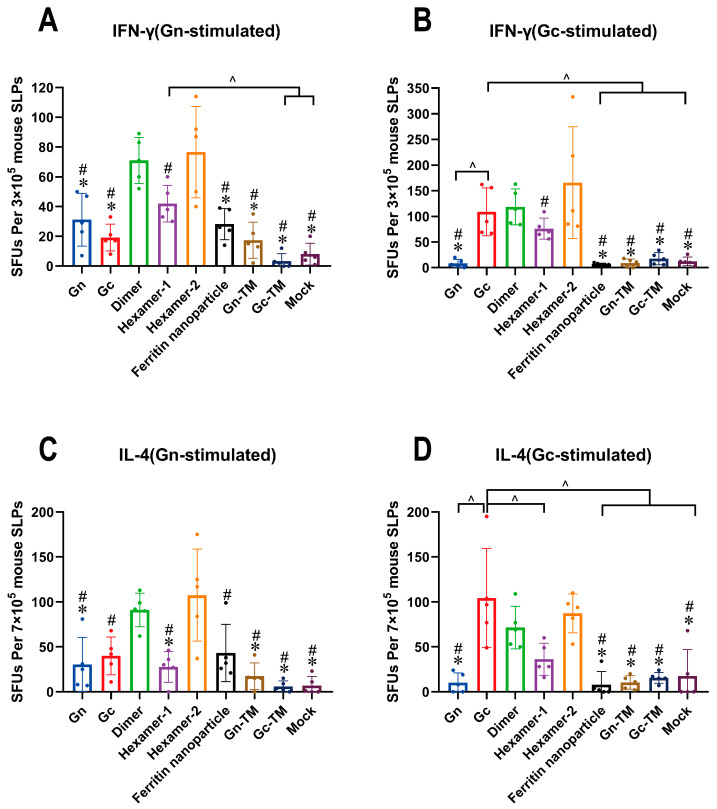
The T cell responses activated by the DNA vaccines. (**A**,**B**) The frequencies of IFN-γ secreting T cells (Th1 pathway) simulated by Gn (**A**) or Gc (**B**) protein at two weeks after the third vaccination detected by ELISpot assay. (**C**,**D**) The frequencies of IL-4 secreting T cells (Th2 pathway) simulated by Gn (**C**) or Gc (**D**) protein at two weeks after the third vaccination detected by ELISpot assay. Mice were treated as described in Figure 2A. *, significantly different from the Dimer group (*p* < 0.05); #, significantly different from Hexamer-2 group (*p* < 0.05); ^, significantly different between the groups indicated in the figures (*p* < 0.05) as determined by one-way ANOVA. Data are presented as mean ± SD (error bars represent SD, *n* = 5 mice per group).

The IFN-γ (Th1 pathway) and IL-4 (Th2 pathway) secreting T helper cells were tested by ELISpot assay after the third vaccination (Figure 4). Gn or Gc protein were used to stimulate the antigen-specific T cells.

For T cells stimulated by Gn, IFN-γ or IL-4-secreting T cells induced by dimer or hexamer-2 were significantly higher than those induced by other Gn-sequence-containing groups except for comparison between dimer and ferritin nanoparticles in IL-4-secreting T cells (Figure 4A,C). For T cells stimulated by Gc, IFN-γ or IL-4-secreting T cells induced by Gc, dimer, or hexamer-2 were significantly higher than those induced by most other Gc-sequence-containing groups (Figure 4B,D).

Specifically, in the Dimer group, the average numbers of IFN-γ or IL-4-secreting T cells induced by Gn stimulation were 71 per 3 × 10^5^ mouse splenocytes (SLPs) and 92 per 7 × 10^5^ mouse splenocytes, respectively. When stimulated by Gc, these average numbers were 114 per 3 × 10^5^ mouse splenocytes and 72 per 7 × 10^5^ mouse splenocytes, respectively. In the Hexamer-2 group, the average numbers of IFN-γ and IL-4-secreting T cells induced by Gn stimulation were 77 per 3 × 10^5^ mouse splenocytes and 108 per 7 × 10^5^ mouse splenocytes, respectively. For Gc stimulation, the average numbers were 165 per 3 × 10^5^ mouse splenocytes and 87 per 7 × 10^5^ mouse splenocytes, respectively. In contrast, the numbers of spot-forming units (SFUs) produced by stimulated T cells in the Gn, Hexamer-1, ferritin nanoparticles, Gn-TM, and Gc-TM groups were relatively low.

In summary, the DNA vaccines of Gc, dimer, and hexamer-2 induced higher T cell responses. The results demonstrating that the DNA vaccines of dimer and hexamer-2 presented strong immunogenicity in both humoral and cellular responses, indicating that they are the dominant constructs among those DNA vaccines.

## 4. Discussion

Since SFTSV was first identified in China in 2009, both its infection cases and geographic distribution have been expanding annually. World Health Organization has designated it as a priority emerging pathogen requiring urgent research. Previous attempts to develop SFTSV vaccines have explored multiple platforms, including inactivated vaccines, recombinant subunit vaccines, viral vector vaccines, and nucleic acid vaccines, with a primary focus on the Gn and Gc glycoproteins as key protective antigens [23]; however, there is still no vaccine available on the market. Among these platforms, inactivated SFTSV vaccines have shown preliminary immunogenicity in animal models, but their development is hindered by biosafety risks during large-scale virus propagation and potential incomplete inactivation [23]. Two recombinant SFTSV-attenuated live vaccines developed from SFTSV HB29 strain by reverse genetics have demonstrated strong IgG and neutralizing antibody responses against both homologous and heterologous SFTSV strains, providing complete protection against lethal SFTSV challenge [24,25]. Viral vector vaccines have shown complete protection against SFTSV in challenge studies using immunodeficient mice, but there is a risk of pre-existing immunity against the vector itself [26,27]. Recombinant subunit protein vaccines offer enhanced safety for immunocompromised people, but the current SFTSV subunit vaccine failed to induce sufficient immunogenicity required for full protection and their immunogenicity is often limited due to the lack of native oligomeric conformations, resulting in insufficient exposure of immunodominant epitopes [28,29]. These challenges, including the risks of genetic-mutation-induced reinfection, excessive immunogenicity of attenuated live vaccines [30,31], and suboptimal immunogenicity with other platforms, highlighted the need for further optimization of antigen design.

Achievements in studies on nucleic acid vaccines promote the development of SFTSV vaccines based on Gn and Gc glycoproteins. Among all SFTSV proteins, Gn and Gc have been confirmed as the most effective antigens for inducing protective immunity, and a DNA vaccine expressing these glycoproteins provided complete protection in aged ferrets against virus challenge [32]. Then, several studies adopted different strategies to enhance the immunogenicity of nucleic acid vaccines. For example, a single plasmid encoding Gn, Gc, NP-NS, Fms-like tyrosine kinase-3 ligand (Flt3L) and IL-12 enhanced cellular immune response and provided complete protection in IFNAR KO mice against lethal SFTSV challenge [33]. Several studies have utilized ferritin nanoparticles with self-assembling properties to load antigens, forming DNA nanovaccine systems, and this strategy enhances the enrichment efficiency of vaccines in target cells and achieves the objectives of targeted delivery and immunomodulation [34,35,36]. For the future development of DNA vaccines, the incorporation of DNA nanovaccine systems may be considered to improve effective immunogenicity. For RNA vaccines, the incorporation of the self-assembling ferritin nanoparticles in a Gn head-based RNA vaccine generated strong binding and neutralizing antibodies and protected mice from SFTSV challenge [37]. Epitopes for cytotoxic T cells or B cells are important in resisting virus invasion. A previous study predicted conservative T and B cell epitopes from Gn, Gc, NP, and Ns and constructed as an mRNA vaccine, which has been confirmed as a potential candidate in inducing immune responses against SFTSV [38]. It can be seen from these studies that both DNA and mRNA vaccines demonstrate advantages in rapid development cycles and cost-effectiveness [33,37,39]. However, the manufacturing process of mRNA vaccines is complex and faces challenges in cold chain transportation, exhibiting lower stability compared to DNA vaccines [32,40]. DNA vaccines are also associated with certain safety concerns. Firstly, there exists a risk that they may persist in the recipient’s cells for an extended period and integrate into the host genome [41,42,43]. Nevertheless, no evidence of plasmid integration has been identified in safety studies on DNA vaccines [44,45]. Secondly, DNA vaccines may trigger non-specific inflammatory responses caused by excessive immune system stimulation, such as autoimmune reactions and allergic responses [44,46]. These issues need to be considered and addressed in future preclinical and clinical research.

These previous efforts have laid the foundation for SFTSV vaccine development but are constrained by key limitations: inadequate recapitulation of viral natural conformations, insufficient cross-genotypic protection, and lack of systematic comparison of antigen structural variants. Addressing these gaps motivated the design of the present study. Given that the complex glycoprotein structures of SFTSV pose significant obstacles for vaccine development, the present study optimized antigen design through constructing Gn and Gc protein structure conformations resembling those on the virus surface based on the DNA vaccine platform by leveraging its advantages in rapid production, high stability, and cost efficiency. These results demonstrated that the heterodimer and heterohexamer of Gn and Gc displayed strong immunogenicity in both humoral and cellular responses, illustrating that the oligomerization of antigens may help enhance their immunogenicity.

The humoral immune responses including binding and neutralizing antibody levels induced by those DNA vaccines increased with each immunization dose compared to the mock group. Among these, Gn and Gc heterodimer, heterohexamer and ferritin nanoparticles induced higher immune responses on average; the neutralizing antibody they induced was also cross-recognized by five other genotype strains (a total of six strains were identified for SFTSV including A–F genotypes) [47,48]. Possibly due to the inter-group variability, neutralizing antibody titer induced by those three groups did not show statistical differences with the mock group after the fourth vaccination. However, the DNA vaccine of Gc, heterodimer, and heterologous hexamer generated robust IFN-γ and IL-4-secreting CD4^+^ T helper cells in the stimulation of Gn or Gc proteins, which were significantly higher than most of the other groups including Gn and ferritin nanoparticles. These results confirm the ability of DNA vaccines to induce potent cellular immune response. This phenomenon may be ascribed to the distinct immunization delivery approach and in vivo mode of action of DNA vaccines compared to other vaccine platforms. We utilized intramuscular injection followed by electrical stimulation to enhance the delivery of DNA vaccine into mouse cells, thereby enabling the vaccine to express immunogens in vivo that are subsequently presented to the immune system via the endogenous antigen processing pathway. Notably, double-stranded DNA can potently activate innate immune signaling; prior studies have documented that DNA vaccine immunogenicity is associated with the STING/TBK1/IFN-αβ axis, as DNA vaccines can engage DNA sensors (e.g., cGAS/STING, AIM2, IFI16, DExD/H-box helicase family members, RNA polymerase III, DAI, DNA-PK, and MRE-11) [49,50,51,52] to indirectly activate the MAPK pathway—a signaling cascade intricately linked to infection, immunity, and inflammation through cellular signal transduction and immune response modulation [53]. Thus, we hypothesize that following intramuscular administration, the DNA vaccine may activate non-specific innate immunity and specific long-term adaptive immunity via the MAPK/ERK pathway, establishing a unique immune microenvironment conducive to protection against infection. Although DNA vaccines administered alone elicit only modest neutralizing antibody titers, their capacity to induce cellular immunity is unparalleled among other vaccine platforms. Currently, numerous research groups employ sequential immunization strategies combining multiple vaccine platforms to achieve complementary advantages; for instance, in the phase 1 clinical trial HVTN 124 evaluating a DNA/protein prime-boost vaccine by Professor Lu Shan’s team, sequential vaccination induced antibody titers exceeding 10,000 in participants, with the elicited functional antibodies predicted to cover over 95% of HIV subtypes and recombinant variants, and all participants mounted high-level CD4^+^ T cell immune responses [54]. Furthermore, HVTN 124 induced functional antibodies with ADCC activity. These observations indicate that the results of our ADCC assay reflect the capacity of the developed DNA vaccine to generate high-quality antibodies, with the Gn and Dimer groups, in particular, eliciting elevated antibody levels. Although the DNA vaccines constructed in this study did not achieve high neutralizing antibody titers and despite the lack of optimized methods to verify the structural integrity of these structure-based antigens, the dominant conformations among those pre-fusion constructs were identified. Utilizing these predominant conformations may enhance the immunogenicity of SFTSV vaccines in further studies, thereby addressing ongoing viral mutations and global public health challenges.

## 5. Conclusions

This study successfully constructed the recombinant DNA vaccines encoding secretory type proteins of monomer and heterologous polymers as well as non-secretory type proteins for Gn and Gc. Through the evaluation of the humoral immunity and cellular immunity induced by those DNA vaccines, this study clarified the following core results: (1) The humoral responses including ADCC activity induced by secretory type of Gn were significantly higher than those induced by its non-secretory counterpart, confirming that the removal of the transmembrane region could improve antigen delivery and immune recognition efficacy; (2) The heterodimer and hexamer-2 (constructed based on the CAT+T4 trimer motif) showed the best performance in humoral immunity (inducing high-titer cross-neutralizing antibodies and strong ADCC activity) and cellular immunity (activating coordinated Th1/Th2 responses), which are identified as the dominant antigen conformations for SFTSV vaccines. The novel protein structures identified in the present study, which induced potent immunogenicity, provided a new strategy in improving the immunogenicity against SFTSV and other bunyaviruses. A key limitation of this study is that current biosafety laboratory regulations and activity restrictions limited the challenge protection tests for those DNA vaccines. Further studies will focus on optimizing the antigen design and exploring alternative vaccine platforms for SFTSV vaccine development.

## Data Availability

The original contributions presented in this study are included in the article. Further inquiries can be directed to the corresponding authors.

## References

[1] Yu X.J., Liang M.F., Zhang S.Y., Liu Y., Li J.D., Sun Y.L., Zhang L., Zhang Q.F., Popov V.L., Li C. (2011). Fever with thrombocytopenia associated with a novel bunyavirus in China. N. Engl. J. Med..

[2] Liu Q., He B., Huang S.Y., Wei F., Zhu X.Q. (2014). Severe fever with thrombocytopenia syndrome, an emerging tick-borne zoonosis. Lancet Infect. Dis..

[3] Zhang X., Liu Y., Zhao L., Li B., Yu H., Wen H., Yu X.J. (2013). An emerging hemorrhagic fever in China caused by a novel bunyavirus SFTSV. Sci. China Life Sci..

[4] Yuan F., Zhu L., Tian D., Xia M., Zheng M.H., Zhang Q., Zhang T., Zhang X., Zheng A. (2024). The first discovery of severe fever with thrombocytopenia virus in the center of metropolitan Beijing, China. Virol. Sin..

[5] You E., Wang L., Zhang L., Wu J., Zhao K., Huang F. (2021). Epidemiological characteristics of severe fever with thrombocytopenia syndrome in Hefei of Anhui province: A population-based surveillance study from 2011 to 2018. Eur. J. Clin. Microbiol. Infect. Dis..

[6] Guo C.T., Lu Q.B., Ding S.J., Hu C.Y., Hu J.G., Wo Y., Fan Y.D., Wang X.J., Qin S.L., Cui N. (2016). Epidemiological and clinical characteristics of severe fever with thrombocytopenia syndrome (SFTS) in China: An integrated data analysis. Epidemiol. Infect..

[7] Woo D., Michelow I.C., Choi Y., Lee H., Park S. (2025). Transmission of severe fever with thrombocytopenia syndrome (SFTS) to humans: A systematic review of individual participant data and meta-analysis. J. Infect. Public Health.

[8] Xu A.L., Xue H., Li Y., Wang X., Zheng J.X., Shi F.Y., Cui Q.X., Lu Y., Cun D.J., Li L.H. (2024). Comprehensive meta-analysis of severe fever with thrombocytopenia syndrome virus infections in humans, vertebrate hosts and questing ticks. Parasites Vectors.

[9] Robles N., Han H.J., Park S.J., Choi Y.K. (2018). Epidemiology of severe fever and thrombocytopenia syndrome virus infection and the need for therapeutics for the prevention. Clin. Exp. Vaccine Res..

[10] Chen Q., Yang D., Zhang Y., Zhu M., Chen N., Yushan Z. (2022). Transmission and mortality risk assessment of severe fever with thrombocytopenia syndrome in China: Results from 11-years’ study. Infect. Dis. Poverty.

[11] Casel M.A., Park S.J., Choi Y.K. (2021). Severe fever with thrombocytopenia syndrome virus: Emerging novel phlebovirus and their control strategy. Exp. Mol. Med..

[12] Kuhn J.H., Adkins S., Alkhovsky S.V., Avsic-Zupanc T., Ayllon M.A., Bahl J., Balkema-Buschmann A., Ballinger M.J., Bandte M., Beer M. (2022). 2022 taxonomic update of phylum *Negarnaviricota* (*Riboviria*: *Orthornavirae*), including the large orders *Bunyavirales* and *Mononegavirales*. Arch. Virol..

[13] Lei X.Y., Liu M.M., Yu X.J. (2015). Severe fever with thrombocytopenia syndrome and its pathogen SFTSV. Microbes Infect..

[14] Yuan F., Zheng A. (2017). Entry of severe fever with thrombocytopenia syndrome virus. Virol. Sin..

[15] Halldorsson S., Behrens A.J., Harlos K., Huiskonen J.T., Elliott R.M., Crispin M., Brennan B., Bowden T.A. (2016). Structure of a phleboviral envelope glycoprotein reveals a consolidated model of membrane fusion. Proc. Natl. Acad. Sci. USA.

[16] Du S., Peng R., Xu W., Qu X., Wang Y., Wang J., Li L., Tian M., Guan Y., Wang J. (2023). Cryo-EM structure of severe fever with thrombocytopenia syndrome virus. Nat. Commun..

[17] Mishra A.K., Hellert J., Freitas N., Guardado-Calvo P., Haouz A., Fels J.M., Maurer D.P., Abelson D.M., Bornholdt Z.A., Walker L.M. (2022). Structural basis of synergistic neutralization of Crimean-Congo hemorrhagic fever virus by human antibodies. Science.

[18] Sun Z., Cheng J., Bai Y., Cao L., Xie D., Deng F., Zhang X., Rao Z., Lou Z. (2023). Architecture of severe fever with thrombocytopenia syndrome virus. Protein Cell.

[19] Liu X., Li Q., Zhang H., Zhang M., Yang Y., Xiao H., He Q., Li H., Wang Y., Li Z. (2025). Gc glycoprotein trimer vaccine elicits robust neutralizing antibodies against severe fever with thrombocytopenia syndrome virus in mice. Int. Immunopharmacol..

[20] Wang C., Yuan F. (2024). A comprehensive comparison of DNA and RNA vaccines. Adv. Drug Deliv. Rev..

[21] Guthe S., Kapinos L., Moglich A., Meier S., Grzesiek S., Kiefhaber T. (2004). Very fast folding and association of a trimerization domain from bacteriophage T4 fibritin. J. Mol. Biol..

[22] Bukreyev A., Camargo E., Collins P.L. (1996). Recovery of infectious respiratory syncytial virus expressing an additional, foreign gene. J. Virol..

[23] Kim D., Lai C.J., Cha I., Jung J.U. (2024). Current progress of severe fever with thrombocytopenia syndrome virus (SFTSV) vaccine development. Viruses.

[24] Yu K.M., Park S.J., Yu M.A., Kim Y.I., Choi Y., Jung J.U., Brennan B., Choi Y.K. (2019). Cross-genotype protection of live-attenuated vaccine candidate for severe fever with thrombocytopenia syndrome virus in a ferret model. Proc. Natl. Acad. Sci. USA.

[25] Hicks P., Manzoni T.B., Westover J.B., Petch R.J., Roper B., Gowen B.B., Bates P. (2024). Safety, immunogenicity, and efficacy of a recombinant vesicular stomatitis virus vectored vaccine against severe fever with thrombocytopenia syndrome virus and heartland bandavirus. Vaccines.

[26] Dong F., Li D., Wen D., Li S., Zhao C., Qi Y., Jangra R.K., Wu C., Xia D., Zhang X. (2019). Single dose of a rVSV-based vaccine elicits complete protection against severe fever with thrombocytopenia syndrome virus. npj Vaccines.

[27] Manzoni T.B., Westover J.B., Lundgreen K.A., Hicks P.D., Petch R.J., Ort J.T., Weissman D., Fan S., Hensley S.E., Pardi N. (2025). Homologous and heterologous vaccination regimens with mRNA and rVSV platforms induce potent immune responses against SFTSV glycoprotein. Viruses.

[28] Liu R., Huang D.D., Bai J.Y., Zhuang L., Lu Q.B., Zhang X.A., Liu W., Wang J.Y., Cao W.C. (2015). Immunization with recombinant SFTSV/NSS protein does not promote virus clearance in SFTSV-infected c57bl/6j mice. Viral Immunol..

[29] Kim D., Kim E., Kim S., Chung Y., Lai C., Cha I., Cho S., Choi Y., Dai X., Kim S. (2023). Self-assembling gn head ferritin nanoparticle vaccine provides full protection from lethal challenge of *Dabie bandavirus* in aged ferrets. MBio.

[30] Pollard A.J., Bijker E.M. (2021). A guide to vaccinology: From basic principles to new developments. Nat. Rev. Immunol..

[31] Kozak M., Hu J. (2023). The integrated consideration of vaccine platforms, adjuvants, and delivery routes for successful vaccine development. Vaccines.

[32] Kwak J.E., Kim Y.I., Park S.J., Yu M.A., Kwon H.I., Eo S., Kim T.S., Seok J., Choi W.S., Jeong J.H. (2019). Development of a SFTSV DNA vaccine that confers complete protection against lethal infection in ferrets. Nat. Commun..

[33] Kang J.G., Jeon K., Choi H., Kim Y., Kim H.I., Ro H.J., Seo Y.B., Shin J., Chung J., Jeon Y.K. (2020). Vaccination with single plasmid DNA encoding IL-12 and antigens of severe fever with thrombocytopenia syndrome virus elicits complete protection in IFNAR knockout mice. PLoS Negl. Trop. Dis..

[34] Wu J., Liang J., Li S., Lu J., Zhou J., Gao M., Zhang Y., Chen J. (2025). DNA nanovaccines derived from ferritin-modified glycogens for targeted delivery to immature dendritic cells and for promotion of Th1 cell differentiation. Acta Biomater..

[35] Qiao Y., Jin S., Nie J., Chang Y., Wang B., Guan S., Li Q., Shi Y., Kong W., Shan Y. (2022). Hemagglutinin-based DNA vaccines containing trimeric self-assembling nanoparticles confer protection against influenza. J. Leukoc. Biol..

[36] Wang B., Li S., Qiao Y., Fu Y., Nie J., Jiang S., Yao X., Pan Y., Zhao L., Wu C. (2022). Self-assembling ferritin nanoparticles coupled with linear sequences from canine distemper virus haemagglutinin protein elicit robust immune responses. J. Nanobiotechnol..

[37] Kim D., Lai C.J., Cha I., Kang S., Yang W.S., Choi Y., Jung J.U. (2023). SFTSV Gn-Head mRNA vaccine confers efficient protection against lethal viral challenge. J. Med. Virol..

[38] Zhu F., Ma S., Xu Y., Zhou Z., Zhang P., Peng W., Yang H., Tan C., Chen J., Pan P. (2025). Development of a novel multi-epitope mRNA vaccine candidate to combat SFTSV pandemic. PLoS Negl. Trop. Dis..

[39] Neeli P., Chai D., Roy D., Prajapati S., Bonam S.R. (2025). DNA vaccines in the post-mRNA era: Engineering, applications, and emerging innovations. Int. J. Mol. Sci..

[40] Papadatou I., Michos A. (2025). Advances in biotechnology and the development of novel human vaccines. Vaccines.

[41] Pagliari S., Dema B., Sanchez-Martinez A., Montalvo Z.G., Rollier C.S. (2023). DNA vaccines: History, molecular mechanisms and future perspectives. J. Mol. Biol..

[42] Wang Z., Troilo P.J., Wang X., Griffiths T.G., Pacchione S.J., Barnum A.B., Harper L.B., Pauley C.J., Niu Z., Denisova L. (2004). Detection of integration of plasmid DNA into host genomic DNA following intramuscular injection and electroporation. Gene Ther..

[43] Ledwith B.J., Manam S., Troilo P.J., Barnum A.B., Pauley C.J., Griffiths T.N., Harper L.B., Beare C.M., Bagdon W.J., Nichols W.W. (2000). Plasmid DNA vaccines: Investigation of integration into host cellular DNA following intramuscular injection in mice. Intervirology.

[44] Silveira M.M., Moreira G., Mendonca M. (2021). DNA vaccines against COVID-19: Perspectives and challenges. Life Sci..

[45] Mori T., Kanda Y., Takenaka K., Okamoto S., Kato J., Kanda J., Yoshimoto G., Gondo H., Doi S., Inaba M. (2017). Safety of asp0113, a cytomegalovirus DNA vaccine, in recipients undergoing allogeneic hematopoietic cell transplantation: An open-label phase 2 trial. Int. J. Hematol..

[46] Silva C.L., Bonato V.L., Dos S.R., Zarate-Blades C.R., Sartori A. (2009). Recent advances in DNA vaccines for autoimmune diseases. Expert. Rev. Vaccines.

[47] Yun S.M., Park S.J., Park S.W., Choi W., Jeong H.W., Choi Y.K., Lee W.J. (2017). Molecular genomic characterization of tick- and human-derived severe fever with thrombocytopenia syndrome virus isolates from South Korea. PLoS Negl. Trop. Dis..

[48] Fu Y., Li S., Zhang Z., Man S., Li X., Zhang W., Zhang C., Cheng X. (2016). Phylogeographic analysis of severe fever with thrombocytopenia syndrome virus from Zhoushan islands, China: Implication for transmission across the ocean. Sci. Rep..

[49] Zahid A., Ismail H., Li B., Jin T. (2020). Molecular and structural basis of DNA sensors in antiviral innate immunity. Front. Immunol..

[50] Suschak J.J., Wang S., Fitzgerald K.A., Lu S. (2016). A CGAS-independent STING/IRF7 pathway mediates the immunogenicity of DNA vaccines. J. Immunol..

[51] Suschak J.J., Wang S., Fitzgerald K.A., Lu S. (2015). Identification of aim2 as a sensor for DNA vaccines. J. Immunol..

[52] Moseman J.E., Shim D., Jeon D., Rastogi I., Schneider K.M., McNeel D.G. (2025). Messenger RNA and plasmid DNA vaccines for the treatment of cancer. Vaccines.

[53] Saggini R., Pellegrino R. (2024). MAPK is implicated in sepsis, immunity, and MAPK is implicated in sepsis, immunity, and inflammation. Int. J. Infect..

[54] Frank I., Li S.S., Grunenberg N., Overton E.T., Robinson S.T., Zheng H., Seaton K.E., Heptinstall J.R., Allen M.A., Mayer K.H. (2024). Safety and immunogenicity of a polyvalent DNA-protein HIV vaccine with matched env immunogens delivered as a prime-boost regimen or coadministered in HIV-uninfected adults in the USA (HVTN 124): A phase 1, placebo-controlled, double-blind randomised controlled trial. Lancet Hiv..

